# Bio-Optimization of Chemical Parameters and Earthworm Biomass for Efficient Vermicomposting of Different Palm Oil Mill Waste Mixtures

**DOI:** 10.3390/ijerph16122092

**Published:** 2019-06-13

**Authors:** Parveen Fatemeh Rupani, Abbas F. M. Alkarkhi, Mohammad Shahadat, Asha Embrandiri, Hany S. EL-Mesery, Hongcheng Wang, Weilan Shao

**Affiliations:** 1 Biofuels Institute, School of Environment and safety Engineering, Jiangsu University, Zhenjiang 212013, Jiangsu, China; hcwang@ujs.edu.cn; 2 Malaysian Institute of Chemical and Bioengineering Technology (MICET), Universiti Kuala Lumpur, Alor Gajah 78000, Melaka, Malaysia; abbas@unikl.edu.my; 3 Department of Biochemical Engineering and Biotechnology, Indian Institute of Technology, IIT Delhi, New Delhi 110016, India; mdshahadat93@gmail.com; 4 Department of Agro-Based Industry, Universiti Malaysia Kelantan, Jeli Campus, Kelantan 17600, Malaysia; ashanty66@gmail.com; 5 School of Agricultural Equipment Engineering, Jiangsu University, Zhenjiang 212013, China; hanyel_mesery@yahoo.com; 6 Department of Crop Handling and Processing, Agricultural Engineering Research Institute, Agricultural Research Center, Dokki, Giza 12618, Egypt

**Keywords:** palm oil mill effluent, palm-pressed fiber, three-dimensional model, optimization, vermicomposting

## Abstract

The present study reports mathematical modelling of palm oil mill effluent and palm-pressed fiber mixtures (0% to 100%) during vermicomposting process. The effects of different mixtures with respect to pH, C:N ratio and earthworms have been optimized using the modelling parameters. The results of analysis of variance have established effect of different mixtures of palm oil mill effluent plus palm press fiber and time, under selected physicochemical responses (pH, C:N ratio and earthworm numbers). Among all mixtures, 60% mixture was achieved optimal growth at pH 7.1 using 16.29 C:N ratio in 15 days of vermicomposting. The relationship between responses, time and different palm oil mill waste mixtures have been summarized in terms of regression models. The obtained results of mathematical modeling suggest that these findings have potential to serve a platform for further studies in terms of kinetic behavior and degradation of the biowastes via vermicomposting.

## 1. Introduction

Rapid industrialization has severely affected the environment leading to increasingly undesired environmental conditions globally. Releasing of wastewaters from various industries into waterbodies is the main cause of environmental pollution. Several countries such as Malaysia has 14% of the land occupied by oil palm plantation [1]. In Malaysia, on average, oil palm plantations, along with palm processing industries, generate approximately 80 million tons of waste by-products, such as palm oil mill effluent (POME) annually [2]. In 2014, the production of POME was estimated to be 44 million cubic meters tons. As a result, huge production of POME became a major challenge to the scientific community and increased further interest to investigate pollutant removal technologies of POME before discharging it into the environment. The effluent and biomass of palm industries have high amount of organic components (approximate chemical oxygen demand (COD) = 80 g/L). Releasing of palm mill wastes to natural water resources causes disturbance of minerals and nutrients plants and algae (hypertrophication) in water [3]. Disturbance of hypertrophication affect chemical oxygen demand (COD) and biochemical oxygen demand (BOD) become a further cause of water pollution [4]. It is found that even a small concentration of industrial wastewater is toxic to the aquatic animals [5]. Therefore, attention has been paid to treat palm mill effluents before discharging to water bodies. For the treatment of industrial effluents, a number of treatment technologies had been used. For the treatment of POME, anaerobic and facultative treatment process in open lagoons was also introduced in the 1970s. However, this treatment caused the emissions of greenhouse gases (e.g., CH_4_, NO_2_, etc.).

### Treatment Technologies of POME and Their Effects to the Environment

Previously, discharging the palm oil mill effluent (POME) into natural water resources (river, lake and sea) was considered as the cheapest way of disposal (since 1970s). However, releasing of POME to the water bodies resulted in water depletion and disturbance of the aquatic environment. In Malaysia, more than 85% of palm oil mills have adopted ponding systems to treat POME [6]. However, open lagoon POME treatment system requires over 10–12 acres of land which is another serious drawback. In addition, other problems are long HRT (hydraulic retention time), fouling, smell and greenhouse gas emissions. Other treatment processes, such as aerobic and anaerobic digestions, physicochemical treatments and membrane filtration technologies, have also been studied separately to treat POME. However, these techniques are either expensive or time consuming.

In most of the palm oil mills around the world, POME has been treated using an anaerobic lagoon system due to its low capital requirement, according to Chin et al. [7]. Hojjat and Salleh, [8] reported that both centrifugation and coagulation methods achieved different pretreatment qualities; however, pretreatment using filtration method was found to be better. Said et. al. [9] observed that adsorption technique followed by ultra filter membrane could effectively treat POME with 98% COD removal. However, the application of these technologies it is often difficult to adhere precisely to the regulations. Thermogravimetric behavior of EFBF (empty fruit bunch fiber) and POME sludge blends (EFBF: POME sludge mass ratio of 100, 90, 75, 50, 25 and 0%) have also been investigated by subjecting it to different heating rates (5, 10, 20, 30 and 40 °C/min), to simulate pyrolysis conditions, in order to optimize production [10].

Apart from different chemical and physical technologies, biological processes such as composting and vermicomposting has become a first focus treatment option in recent research [11,12]. This is because of its rapid biologically processed mechanism to produce value added product (biocompost and fertilizers). Baharuddin et al. [13] used co-composting to utilize POME and shredded fiber. Additionally, the maturity of the composting material can be achieved in 40 days with C:N ratio below 20. Rupani et al. [14] studied the applicability of acidic POME using vermicomposting technology (2012) and successfully achieved matured compost (C:N of 14) in 40 days. Hayawin [15] investigated the viability of vermicompost cultivated in a media comprising of oil palm empty fruit bunch (OPEFB) and anaerobically digested palm oil mill effluent (POME). Palm oil seedling treated in POME:OPEFB (50:50) was germinated greater than frond, stem and root (dry weight), compared to being solely treated with chemical fertilizers.

Further experiments was conducted by Rupani et al. [16] to evaluate optimal vermicompost of three selected mixture of POME-PPF (palm-pressed fiber) (50%, 60% and 70%). They showed that 50% POME-PPF mixture was found as the most suitable vermicompost in terms of earthworm and plant growth criteria. Rupani et al. [15] confirmed their results by the evaluation of decomposition rate through kinetic study and reported that it follows first order reaction, while the 50% mixture achieved higher decomposition rate (*k*50% = 0.0498 day^−1^) (following the method of Levenspiel [17] as first-order kinetics with the rate constant (*k*) using the following equation $n\;\left(\frac{{\left[A\right]}_{t\;}}{{\left[A\right]}_{0\;}}\right)=-kt\;$; where [*A*] is the concentration at time *t* and [*A*]o is the concentration at time *t* = 0, and *k* is the first-order rate constant (day^−1^)). However, detailed kinetic evaluation of biodegradation study of different combination of POME-PPF during vermicomposting process is still in its nascent stage. To date, no further study has been conducted to evaluate the mathematic modelling of POME-PPF mixtures to analyze its behavior with respect to different C:N ratios over time during vermicomposting process. Therefore, in the present study, mathematical modelling of different POME-PPF mixtures along with its behavior towards pH, C:N ratio changes and earthworm growth during vermicomposting process were evaluated. From this work, an optimized mixture for vermicomposting can be selected.

## 2. Materials and Methods

### 2.1. Materials

Fresh palm oil mil effluent (POME) and palm-pressed fiber (PPF) were obtained from a palm oil mill located in Penang, Malaysia. Earthworms belonging to *Eudrilus eugeniae* species were collected from the vermiculture unit (Penang, Malaysia). The precultured earthworm was maintained in plastic containers with partially decomposed mixture of biowaste (as a growth medium) under laboratory conditions (26 ± 2 °C) for further use.

### 2.2. Experimental Set-Up

The precomposting experiment was set up using vermireactors (rectangular plastic container with actual volume of 144 cm^3^). In order to drain water from the reactor, equal holes were created at the bottom of each reactor. The fixed amount (50 g) of homogenized POME and PPF mixtures in different combinations (100, 90, 80, 70, 60, 50, 40, 30, 20, 10 and 0% of POME) were selected and remaining percentages of PPF (on the basis of wet weight) were placed in each reactor. All the vermireactors were set in triplicates with a total of 33 experimental units. The present research focuses on the efficiency of the earthworm in different mixtures; hence, each reactor was offered as a comparison study for the other.

**Figure 1:**
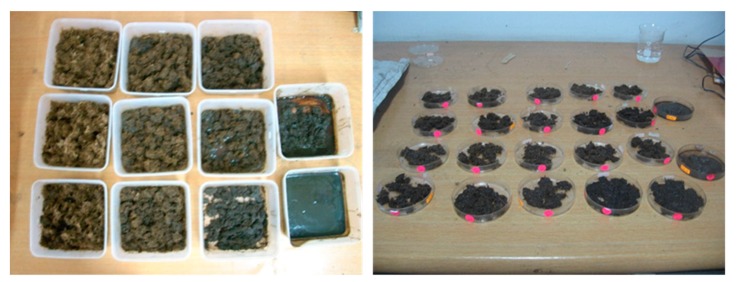
(**left**) Pre-composting phase for different POME-PPF mixtures (0%, 10%, 20%, 30%, …, 100%) (*n* = 3 for each treatment). (**right**) Vermicomposting phase for different percentages of POME-PPF mixtures in Petri dishes (*n* = 3 for each treatment).

Adequate quantities of distilled water were added to each reactor in order to keep the substrate moist. After stabilizing (precomposting) the mixture in plastic containers, the vermicomposting process was carried out using the Petri plate method to observe the results in shortest time duration. Hence, after five days of precomposting, 10 g sample from each mixture was placed in a Petri dish and five clitellate earthworms of *E. eugeniae* species (each of 3.10 ± 1 g) were added to each container to evaluate the vermicomposting process within 10 days. Petri dishes were positioned in a dark place at controlled laboratory condition (25 ± 2 °C). Figure 1 shows the experimental documentation. The pH variation, carbon to nitrogen changes and the growth of earthworms was monitored in each experimental reactor. At every two-day interval, the earthworms were separated from each Petri dish, washed in tap water to remove the adhering material from their body and subsequently weighed. The weighed earthworms were returned to their respective Petri dishes. As expected, the earthworms could not survive in 100% POME, hence data pertaining to 100% POME was not studied in the present research.

The collected samples (in the absence of earthworms) were oven dried at 105 °C, ground and stored in labelled plastic bags for further analysis using recommended standard methods. The pH of vermicompost was determined using a double-distilled water suspension in the ratio of 1:10 (w/v) using pH Meter (Hach Sension 3). The amount of total organic carbon (TOC) and nitrogen (N) were measured by CHN analyzer (Shimadzu Model 2400) [18].

### 2.3. Statistical Analysis and Modelling

Data obtained from different experiment were analyzed using analysis of variance (ANOVA) to study the effect of pH, C:N ratio and earthworms’ growth. Furthermore, mathematical regression models were applied using MATLAB software (MATLAB, 2000, The MathWorks, Natick, MA, USA) to summarize the relationship among different POME-PPF mixtures along with three variables (pH, C:N ratio and earthworms) with respect to time [19].

## 3. Results and Discussion

Carbon and nitrogen are two primary nutrients required for cell growth, therefore, an optimal carbon-to-nitrogen ratio is necessary for vermicomposting [20]. This study was carried out to evaluate how different mixtures of POME-PPF (percentages) and times (0, 5, 10 and 15 days) could affect the C:N ratio, pH and earthworm biomass during vermicomposting. Previously, Komolis [21] studied the optimization of dewatered sludge. The authors have evaluated the optimal mixing ratios of dewatered sludge with other organic amendments to enhance the degradability of the mixtures during composting. Authors have generated a mathematical model based on the experimental design procedure to quantify the contribution of each organic component in the biodegradability of the whole mixture. Prioeti [22], generalized a mathematical formulation of chemical–physical, as well as economic, constraints that recur in composting mixture design. The main aim of their study was to implement a mathematical model by defining the functional characteristics of a software application, which take into account simultaneously the economic variables, the physicochemical properties and the operational constraints. According to the authors, this information could be required for a good start of the composting processes. Therefore, in the present study, mathematical modelling of different POME-PPF mixtures, along with its behavior towards pH, C:N ratio changes and earthworm growth during vermicomposting process, were evaluated through determining which optimized mixture for vermicomposting can be selected for scale up of this technology on pilot plant. To date, no mathematical model has been generated to predict the optimized mixture of different palm oil mill ratio.

The initial characteristics of POME and PPF were conducted in our previous study [23]. The results reported POME had the initial chemical characteristics of pH 3.9, C (31.5%) and N (3.8%), while PPF had initial values of pH 7.18, C (45.21%) and N (0.5%). 

### 3.1. Effects of pH, C:N Ratio and Earthworm Biomass during the Vermicomposting Process

The time duration and the composition of substrates are main parameters that influence the variation of pH, C:N ratio and earthworm biomass during the vermicomposting process. Ten different percentages of POME-PPF mixture (0, 10, 20, 30, 40, 50, 60, 70, 80 and 90%) were examined over 0, 5, 10 and 15 days. The data obtained from the different experiments were subjected to ANOVA in order to test whether the selected variables (percentage of POME-PPF mixtures and time) have significant effect on pH, C:N ratio and earthworm biomass during vermicomposting. The results provided a better understanding as to the response of pH, C:N ratio and earthworm biomass at different levels (setting) for the selected parameters. The results of ANOVA (Table 1) showed that the two parameters (time and the percentage of POME-PPF) were both significantly important and influenced the pH (*p* < 0.0001), while the interaction between time and the percentage of POME-PPF was insignificant, indicating that time and POME-PPF percentage affect the pH value independently.

Different percentages of POME-PPF mixture was affected by either increasing or decreasing the pH. Improvement in the pH of POME-PPF mixture could be due to the release of ammonia gas after protein degradation [24,25]. Other POME-PPF mixtures also showed a decline in pH value at the end of vermicomposting, which could be due to the decomposition of organic content and further transformation into the ammonium ions and humic acids [26]. However, alternation in pH values might be due to the rapid metabolic degradation of organic acids, as well as intense proteolysis of alkaline ammonia during the degradation of protein [27,28].

The effect of time and the percentage of POME-PPF mixture on C:N ratio were also studied. The results of ANOVA (Table 1) established that time and different percentage of POME-PPF mixture had significant influence on C:N ratios (*p* < 0.0001). A reduction in the C:N ratio during the vermicomposting was observed which might be due to the improvement in nitrogen level and decline in the level of total carbon in the mixture. Enhancement in nitrogen level is attributed to the degradation of organic compounds which subsequently released CO_2_ in the mixture [29]. Moreover, the activity of earthworms during vermicomposting process led to the release of ammonia via excretion [30].

In addition, the growth of *E. eugeniae* was significantly influenced by the time and percentage of POME-PPF (*p* < 0.0001, Table 1), while the interaction between time and the percentage of POME-PPF was insignificant indicating that time and percentage of POME-PPF affect the earthworm growth independently. The results established that earthworm biomass increased in 40, 50, 60 and 70% of POME-PPF mixtures. Decline in earthworm biomass could be due to the food shortage in the vermibeds [31]Furthermore, a decrease in earthworm biomass was attributed to the acidic condition of the substrate while the percentage of POME was increased in the mixture. The earthworms did not survive in 100% POME-PPF mixture as a result of its high acidity.

### 3.2. Mathematical Modelling of POME-PPF with Respect to pH, C:N Ratio and Earthworms

**Figure 2:**
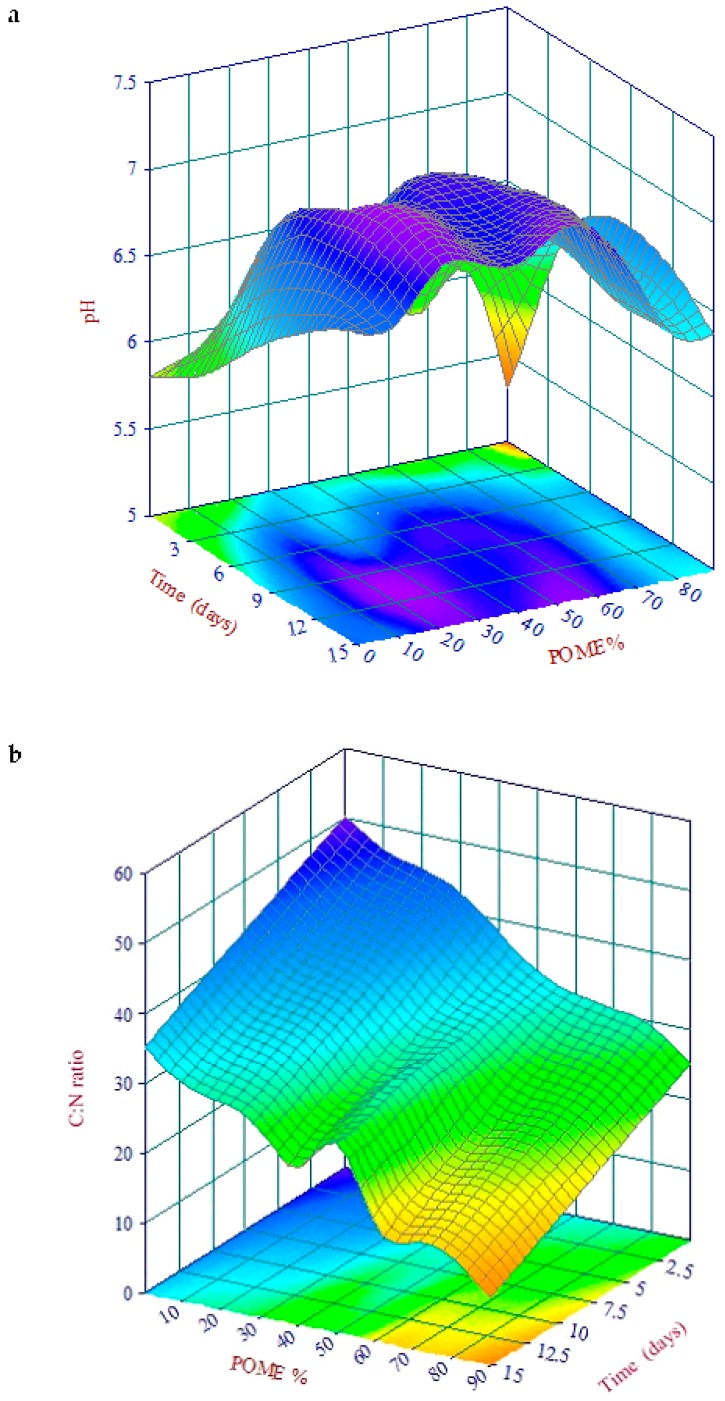

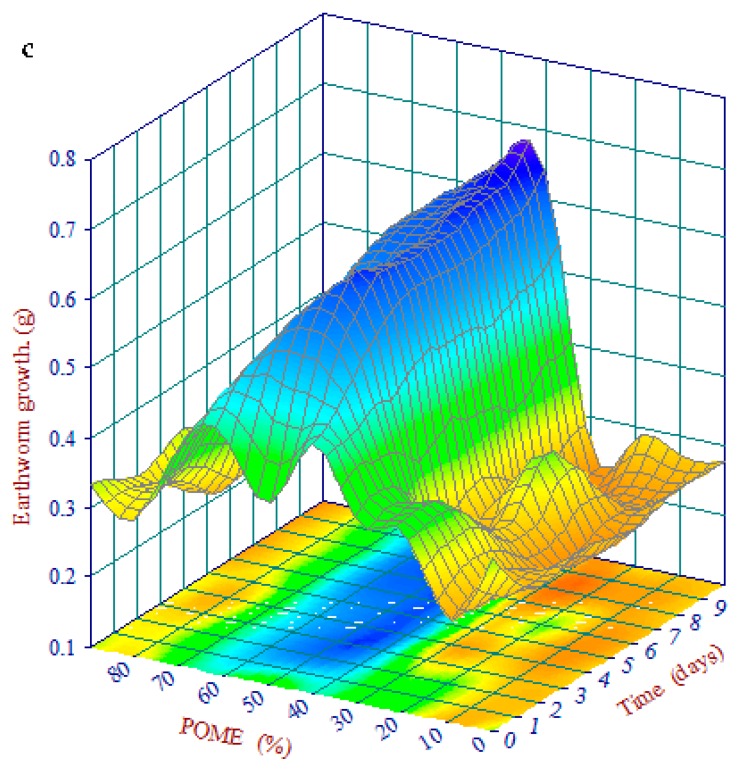
Three-dimensional response surface plot of (**a**) pH, (**b**) C:N ratio and (**c**) EW.

The mathematical models were generated in order to understand the behavior of earthworm growth over different time and percentage of POME-PPF mixtures in the vermicompost. Figure 2 represents the pH value for different percentages of POME-PPE and time. Multiple regression analysis for the interaction effect of POME-PPF and time can be represented by Equation (1)

(1) $$Z=a+b\ln x+cy+d{\left(\ln x\right)}^{2\;}+ey^{2}+fy\ln x+g{\left(\ln x\right)}^{3}+hy^{3}+iy^{2}\ln x+jy{\left(\ln x\right)}^{2}$$

R^2^ = 0.914; where
Z = pH values; x = Time (days); y = POME (%);
*a* = 4.038; *b* = 1.87; *c* = 4.24; *d* = −0.32; *e* = −4.72; *f* = −0.59;
*g* = −0.00047; *h* = 1.49; *i* = 0.00067; *j* = −0.00085.

A three-dimensional response surface plot to have a clear picture of the behavior of time and mixture of pH is shown in Figure 2a.

Multiple regression analysis was used to determine the relationship between C:N ratio, POME-PPF and time. The polynomial regression equation and the associated coefficient of determination (R^2^) are shown in Equation (2); Figure 2b.

(2) $$Z=a+bx+cx^{2}+dx^{3}+ey+fy^{2}+gy^{3}+hy^{4}+iy^{5}$$

R^2^ = 0.9785; where
Z = C:N; x = Time (days); y = POME (%);
*a* = 50.22; *b* = −1.066; *c* = 0.0145; *d* = −0.00056; *e* = −0.636; *f* = 0.032;
*g* = −0.00098; *h* = 1.24; *i* = −5.56.

From the results of multiple regression analysis (Figure 2c), earthworm growth can be calculated using Equation (3), based on the POME time

(3) $$Z=a+bx/\ln x+cy\ln y+dy^{1.5}+ey^{2}+fy^{2}\ln y+gy^{2.5}+he^{y/wy}$$

R^2^ = 0.8123; where
Z = earthworm growth (g); x = Time (days); y = POME (%);
*a* = 8966.44; *b* = 0.027; *c* = 2.95; *d* = −6.58; *e* = 1.621; *f* = −0.296;
*g* = 0.0227; *h* = −8967.75; *wy* = −2294.70.

Equation (2) describes the behavior of C:N ratio at different time and mixture of POME. The C:N ratio of the vermicompost reflected the mineralization and stabilization of the substrate. The earthworms assist in addition of nitrogen in the form of mucus and nitrogenous excretory material and decreased carbon content through releasing of carbon dioxide [32]. The process of vermicomposting causes reduction in C:N ratio in the mixture. The three-dimensional response surface plot is used to show pH as a function of time and POME percentage is shown in Figure 2a. The C:N ratio affected the earthworm growth directly, as reported by Rupani et al. [33] that high C:N ratio positively increased the earthworm biomass. Equation (3) explains the behavior of earthworm biomass at different times, shown in Figure 2c.

### 3.3. Optimization of Vermicompost

Regression models have been developed for pH, C:N ratio and earthworm biomass in order to select the best POME-PPF mixture to achieve the highest earthworm growth with best C:N ratio at the end of vermicomposting (day 15). Once the models have been built and checked for adequacy, the optimization criteria can be set to determine optimum operating conditions. Based on achieved results, optimized mixture of POME was found to be 15 days of vermicompost process in 60% POME-PPF mixture which resulted in pH 7.1, C:N = 16.2933 and earthworm = 0.446667. A confirmatory was carried out by the software to validate the obtained regression model. Hence, the results confirmed the best fit at pH 7, C:N = 16.3352 and earthworm = 0.451.

## 4. Conclusions

The present study exposed two parameters (time and the percentage of POME-PPF) which significantly influenced the value of pH, C:N ratio and, consequently, the effect on the earthworm biomass during vermicomposting. The findings of vermicomposting time independently affected the POME-PPF mixture characteristics. The results from optimization criteria through regression models indicated that 60% POME-PPF mixture was found to be the best fitted mixture along with pH 7.1, C:N = 16.2933 and earthworm = 0.446667 in 15 days of vermicomposting. A 3D model generated from the mathematical model can be used to predict the C:N ratio and earthworm biomass with respect to time in different POME-PPF mixtures; however, the finding of this research is limited to short-term vermicomposting process. The driving force behind decomposition and organic matter formation is the microbial utilization of residues for the production of metabolic energy and growth. Many reactions occurring during decomposition of organic matter are slow, and kinetic considerations are, therefore, important. Hence, it would be interesting to repeat the experiment and evaluate the C:N ratio changes and earthworm growth performance along with its microbial changes during the process and generate the mathematical model predictions which could be applied for vermicomposting of any kind of organic wastes.

## Figures and Tables

**Table 1:** The results of ANOVA for pH, C:N and earthworm (EW) values during the vermicomposting of different POME percentages in PPF (*Eudrilus eugeniae*).

S.O.V.	S.S.	d.f.	M.S.	F	*p*-Value
(pH)					
Model	24.33	39	0.62	4.47	<0.0001
A-Day	16.03	3	5.34	39.11	<0.0001
B-Mixture	5.78	9	0.64	4.70	<0.0001
AB	2.51	27	0.093	0.68	0.8685
Pure Error	10.93	80	0.14		
Total	35.26	119			
(C:N)					
Model	11,421.86	39	292.87	216.82	<0.0001
A-Day	3516.43	3	1172.14	867.78	<0.0001
B-Mixture	7808.85	9	867.65	642.35	<0.0001
AB	96.57	27	3.58	2.65	0.0004
Pure Error	108.06	80	1.35		
Total	11,529.92	119			
(EW)					
Model	2.78	39	0.071	42.76	<0.0001
A-Day	2.22	3	0.74	444.54	<0.0001
B-Mixture	0.29	9	0.032	19.19	<0.0001
AB	0.27	27	0.009947	5.98	<0.0001
Pure Error	0.13	80	0.001668		
Total	2.92	119			

S.O.V.: Source of variation; S.S.: Sum of square; d.f.: Degree of freedom; M.S.: Mean of sum of square; F: F-value; and *p*-value.
